# Risk of burnout in French entrepreneurs during the COVID-19 crisis

**DOI:** 10.1007/s11187-021-00516-2

**Published:** 2021-06-11

**Authors:** Olivier Torrès, Alexandre Benzari, Christian Fisch, Jinia Mukerjee, Abdelaziz Swalhi, Roy Thurik

**Affiliations:** 1 Université de Montpellier, Montpellier, France; 2 Montpellier Business School, Montpellier, France; 3 Montpellier Research in Management, Université de Montpellier, Montpellier, France; 4 Universität Trier, Trier, Germany; 5 Erasmus School of Economics, Rotterdam, Netherlands

**Keywords:** Small business owners, Entrepreneurs, Burnout, COVID-19 pandemic, France, I30, L26

## Abstract

**Abstract:**

The COVID-19 crisis presents manifest threats for entrepreneurs since their business survival is often directly at stake given the alarming economic downturn. This existential threat, together with their crucial role in the economy, is the reason for the plethora of public financial support schemes being implemented throughout the entire world. However, support schemes for mental health are lacking. We aim to investigate, *first*, whether burnout levels have increased during the crisis and, *second*, whether burnout levels during the COVID-19 crisis depend on the threat of becoming ill, having to stay at home due to the lockdown, and/or having to file for bankruptcy due to the economic downturn. We do so using seven data sets of French entrepreneurs with a temporal comparison of averages and two data sets of French entrepreneurs with a cross-sectional analysis of individuals. Our findings show that indeed, the risks of burnout have increased during the pandemic and that the threat of bankruptcy is the dominant threat. As an increasing number of studies in the entrepreneurship literature indicate that entrepreneurs’ mental health influences their activities, as well as the growth and sustainability of their ventures, our study is important and timely in its contribution, as it takes a close look at the perception of burnout in general and more specifically during the COVID-19 pandemic.

**Plain English Summary:**

The risk of burnout in French entrepreneurs has increased significantly during the COVID-19 pandemic, which calls for not only financial support but also other forms of support. The COVID-19 pandemic presents many threats for entrepreneurs since their business survival is often directly at stake. These threats are not just financial but also related to health, such as the threat of burnout. The findings of our study show that for French entrepreneurs, the threat of burnout increased after the arrival of the COVID-19 pandemic. This finding raises the question whether this outcome is due to the threat to health, the effects of the lockdown, or the threat of bankruptcy. It appears that all three factors play important roles, although the financial threat is the dominant threat. These findings call for the extension of entrepreneurial support systems beyond the financial area by also involving an “entrepreneurship care” aspect, which includes telephone support, webinars, and mental help facilities.

## Introduction

Burnout is a threat to any job or profession. Entrepreneurs^1^ are no exception. Burnout is a “state of physical, emotional, and mental exhaustion caused by a long-term involvement in situations that are emotionally demanding” (Pines & Aronson, 1988: p. 9). It was first documented in the 1970s among social and health care workers (Freudenberger, 1975; Maslach, 1976). Since then, burnout has slowly become a ubiquitous phenomenon. In 2014, Maslach and Leiter, the founders of the journal *Burnout Research*, observed that more than a thousand scientific articles are published annually on the subject of burnout in more than a hundred scholarly journals (Maslach & Leiter, 2014). In May 2019, the World Health Organization recognized burnout as an “occupational phenomenon,” and this definition was included in the 11th Revision of the International Classification of Diseases (World Health Organization, 2019).

However, the burnout of entrepreneurs has only recently become a topic of interest (Jamal, 2007; Perry et al., 2008; Shepherd et al., 2010; Hatak et al., 2015; Wei et al., 2015; Fernet et al., 2016; Mol et al., 2018; Omrane et al., 2018; Soenen et al., 2019; Torrès, Benzari, et al., 2021b, forthcoming). In part, this is because non-salaried workers are not part of the public occupational health system in various countries. However, entrepreneurs are particularly susceptible to burnout compared to salaried employees (Kuan-Han et al., 2020) since they work longer hours (Bodier et al., 2010), spend less time on leisure activities (Van der Zwan et al., 2018), sleep less (Guiliani & Torrès, 2018; Gunia, 2018), and have a more stressful working life due to their economic and managerial responsibilities (Dahl et al., 2010).

While the causes for the burnout of entrepreneurs and salaried employees may not differ, the consequences are largely different due to their divergent economic and social roles. Even the smallest health issue of an entrepreneur can have repercussions for the entire business (Chao et al., 2007), particularly if the business is small. Mintzberg described small businesses as “... hinging on the health and whims of one individual. One heart attack can literally wipe out the organization’s prime coordinating mechanism” (Mintzberg, 1979: p. 312). Thus, the health of the business owner is perhaps the foremost intangible capital of a small business (Stephan, 2018; Torrès, 2012; Torrès & Thurik, 2019).

The COVID-19 epidemic is bound to affect the mental health of entrepreneurs (Cullen et al., 2020; Pfefferbaum & North, 2020), as well as their burnout level (Yildirim & Solmaz, 2020). In the present study, we distinguish between three main reasons for a COVID-19-induced increase in burnout. The *first* is the general risk of catching the COVID-19 virus, which incurs illness and/or quarantine, and (sometimes) hospitalization. The *second* is related to the consequences of periods of lockdown in industries that make working from home difficult. The *third* is related to downsizing, business failure, and even involuntary exit due to the unprecedented slowdown of economic activities in most industries in 2020 and 2021.^2^

Our study deals with two specific questions. First, has the perceived burnout level of entrepreneurs increased during the pandemic? Second, how can these levels be explained during the crisis, in particular by discriminating between health risks (proxied by the threat of becoming ill), the effect of the lockdown (proxied by the degradation of the quality of life specifically due to lockdown), and/or the influence of perceptions of economic risks (proxied by the threat of having to file for bankruptcy)?

We do so by using a unique series of seven data sets of French entrepreneurs. We use all seven datasets to investigate average burnout levels over time, that is, before and during the COVID-19 pandemic. In total, these data sets comprise five thousand individuals. Then, we perform analyses in which we use two of these data sets to establish the links between perceptions of individual burnout levels, the threat of illness, the effects of lockdown, and the threat of bankruptcy, while controlling for many variables. Our results show that average burnout levels are indeed higher during the COVID-19 pandemic than before and that these burnout levels are connected to all three effects (health risks, effects of the lockdown, and economic risks), with economic risks (bankruptcy) having the largest effect.

Our study contributes to the research on the general mental health of entrepreneurs (Omrane et al., 2018; Stephan, 2018; Torrès & Thurik, 2019) and focuses on burnout in particular. The advent of the COVID-19 pandemic allows us to investigate changes in perceptions of burnout and their causes. Discriminating between three causes (i.e., the threat of illness, the effects of lockdown, and the threat of bankruptcy) is a novel setup that enables us to distinguish the different roots for increase in burnout in a nuanced way. Since some of these risks are specific to the COVID-19 crises, while others are not, our findings enable future research to better differentiate crisis-specific and time-constant determinants in increasing burnout levels. Hence, our work is embedded in the risk perception literature. Risk perception towards COVID-19 and its understanding is an important topic (Van Bavel et al., 2020). There is a current call in the literature for understanding the “new vulnerabilities [of entrepreneurs] that diminish their psychological wellbeing” (Shepherd, 2020: p. 3). By looking at two kinds of risk perception (risk perception being the appraisal of danger or threat (Dryhurst et al., 2020)) for entrepreneurs during COVID-19, we show how the perception of both health and economic risks linked to COVID-19 endanger entrepreneurs’ mental health and leads to the risks of burnout. In formulating our hypotheses, we use concepts from Stress Event Theory, the Job demand -resource model, and the Conservation of Resource Theory.

The present paper is organized as follows. Section 2 deals with a concise review of the literature. The data, variables, and measurements are discussed in Section 3, while the results are presented in Section 4. In Section 5, the results are interpreted, while Sections 6 and 7 consider limitations and future research avenues and recommendations, respectively.

## Short literature review and hypotheses

### Burnout and the environment

While the literature on stress emerged during the mid-twentieth century, based on the work of Selye (1956) and Lazarus (1966), entrepreneurial stress and burnout is a relatively recent research topic (Boyd & Gumpert, 1983; Lerman et al., 2020; Omrane et al., 2018; White & Gupta, 2020). Stress can be defined as “a particular relationship between the person and the environment that is appraised by the person as taxing or exceeding his or her resources and endangering his or her well-being” (Lazarus & Folkman, 1984: p. 19).

Various studies have identified stressors of entrepreneurs. Fernet et al. (2016) identified five sources of stress/stressors, namely, human resource management, sales, finances, supplies, and administration. Recently, based on a systematic literature review, White and Gupta (2020) identified three major sources of stress for entrepreneurs, namely, role-related stress, stress related to business operations, and general life stress. Role-related stress refers to the different role stressors, such as role ambiguity, role conflict, and role overload, which are experienced by entrepreneurs (see role theory of Kahn et al., 1964). The stress related to business operations refers to the work-related responsibilities of entrepreneurs. Finally, general life stress refers to the stress experienced by entrepreneurs outside of the work environment (White & Gupta, 2020). Thus, on a daily basis, entrepreneurs experience stress due to their role and their responsibilities at work, as well as their personal life. These stressors can become a source of burnout when there is an imbalance between the demands of their environment and their existing resources (Wei et al., 2015).

The advent of the COVID-19 pandemic has created yet another array of stress for entrepreneurs. Recent studies have identified stress stemming from the perceived threat of infection and financial problems (this being more acute during the pandemic), which can lead to loneliness, work-home conflict, insomnia, fear, confusion, and anger (Brooks et al., 2020; Bu et al., 2020; Coyne et al., 2020; Rossi et al., 2020; Trnka & Lorencova, 2020). These additional stressors are likely to affect the mental health of entrepreneurs (Cullen et al., 2020; Pfefferbaum & North, 2020) and increase their levels of burnout (Yildirim & Solmaz, 2020).^3^

To understand the additional sources of stress caused by the COVID-19 pandemic, we conceptualize the pandemic as a stress-inducing event that can have negative consequences on entrepreneurial health (Cardon & Patel, 2015). The stress events theory (SET) which was developed by Lerman et al. (2020) and is based on previous theories such as appraisal theory (Lazarus, 1966), conservation of resources theory (Hobfoll, 1989), and event-based system theory (Morgeson et al., 2015) suggests that entrepreneurs interpret and react to events based on event characteristics (strength and duration), the accuracy of interpretation, and developed resources and appraisal coping mechanisms. These interpretations and coping processes of events can then lead to various consequences. However, what is important to note is that it is not the event but rather the perception of it that is consequential (Lerman et al., 2020). The theoretical lens of SET can be useful to understand entrepreneurial behavior during the event of the COVID-19 pandemic, particularly the entrepreneurs’ perception of burnout.

Indeed, the additional source of stress caused by the COVID-19 pandemic can also be conceptualized using the lens of both Conservation of Resource Theory (COR) (Hobfoll, 1989) and the Job-Demand-Control (JDR) model (Demerouti et al., 2001). According to the COR theory (Hobfoll, 2001), all individuals are motivated to retain, protect, and acquire things that are valuable to them, referred to as “resources” (Hobfoll, 2002). When not confronted by stressors, people develop resource surpluses, a “resource reservoir.” On the other hand, when confronted by stressors, resources are depleted. In the context of the pandemic, the additional stressors faced by entrepreneurs, stemming from the perceived threat of infection, financial problems, social isolation, etc., are likely to deplete their resources. Since according to the COR, resource loss is more salient than resource gain (see Hobfoll (2001) for details on resource gain and resource loss spirals), this incongruence between resource gain and resource loss can lead to emotional exhaustion, and increase the perception of burnout in entrepreneurs.

The JDR model is also a framework that can explain mental exhaustion of entrepreneurs during the COVID-19 pandemic. According to the JDR model, job demands are those conditions that require intensive physical, mental, or emotional efforts and thus lead to physical and/or mental costs. Excessive demands act as stress, can lead to physical and mental exhaustion, and adversely affect mental and physical health (Demerouti et al., 2001). Job resources, on the other hand, refer to the physical, psychological, and social aspects of a job that helps reduce the demands and are functional and necessary to deal with job demands and achieved work goals.

According to the JDR model, there are occupational specificities regarding factors that can lead to work-related stress. In fact, prior entrepreneurship research indicates that entrepreneurs have different work characteristics compared to salaried workers—like higher levels of risk, uncertainty, responsibility, and workload, which acts as stressors, and can be considered as higher demands (Collins et al., 2004; Dijkhuizen et al., 2014; Drnovsek et al., 2010; Gorgievski & Laguna, 2008; Tetrick et al., 2000). This means that entrepreneurs generally experience higher levels of stress than salaried workers. We contend that the array of additional stressors specific to the pandemic for entrepreneurs, lead to higher levels of demands than the available resources. Such an imbalance between demands and resources is likely to increase entrepreneurs’ level of burnout.

Thus, we propose the following hypothesis:

- H1: The average perception of burnout in entrepreneurs is higher during the COVID-19 crisis than it was before.

### Health risk perception and burnout

The perceived risk of being infected by COVID-19 can be consequential for the mental health of individuals. As studies indicate, this can lead to a decline in the perception of the level of mental health (Cullen et al., 2020; Ornell et al., 2020; Pfefferbaum & North, 2020) and burnout (Giusti et al., 2020; Hu et al., 2020; Yildirim & Solmaz, 2020). This relationship has been shown for health professionals and nurses (Giusti et al., 2020; Hu et al., 2020), as well as for adults in general (Yildirim & Solmaz, 2020).

Entrepreneurs are salient to their business; thus, their (ill) health not only impacts them personally but also endangers the existence of their business and the livelihood of their employees. Based on SET (Lerman et al., 2020), perceived health risk during the COVID-19 pandemic (the latter being an event) can be conceptualized as a source of stress for entrepreneurs, which can lead to a decline in the perception of their mental health and consequently to burnout. However, to our knowledge, no studies have investigated the link between the perception of health risk and burnout for entrepreneurs during the COVID-19 pandemic. Thus, we propose the following hypothesis:

- H2: The health risk perception of entrepreneurs is positively related to their perception of burnout.

### Lockdown and burnout

In addition to the health risks related to COVID-19, one cannot ignore the effects of the lockdown. In France, the first lockdown in 2020 was effective between 17 March and 11 May 2020. Our surveys were taken during this lockdown period. There are probably many ways in which the lockdown affected individual’s perception of mental health and burnout. For entrepreneurs, lockdown imposed virtual work from home, led to an array of business operation constraints, and in many cases, led to temporary shutdown of businesses. Working from home (resulting from the lockdown) could have also led to an array of demands (lack of work space, increased responsibilities at home due to children not going to school, social isolation, etc.).^4^ The lockdown likely led to frustration, loneliness, and worries about the future, which are also risk factors for mental illness (Banerjee & Rai, 2020; Giallonardo et al., 2020). In fact, a recent review of the impact of quarantine showed that most studies report negative psychological effects, including posttraumatic stress symptoms, confusion, and anger (Brooks et al., 2020). Most of these effects resulted from imposed restrictions on liberty; on the other hand, voluntary quarantine led to less distress (Brooks et al., 2020).^5^

- H3: The effects of the lockdown are positively connected to the perception of burnout in entrepreneurs.

### Economic risk perceptions and burnout

The COVID-19 pandemic has had a serious financial impact on small businesses (Bartik et al., 2020; Block et al., 2021; OECD, 2020) and entrepreneurs (Fairlie, 2020) due to a sharp decline in business activities. This has led to (partial) layoffs and temporary furloughs (Bartik et al., 2020).

Financial problems have a negative effect on the health and well-being of entrepreneurs (Annink et al., 2016; Lechat & Torrès, 2016). Studies indicate that financial problems and the threat of bankruptcy can lead to entrepreneurial burnout (Fernet et al., 2016) and even suicidal tendencies (Bah & Gaillon, 2016; Bortolussi, 2012; Kaneko et al., 2011). The fear associated with financial problems and the threat of bankruptcy could stem from a sense of responsibility that entrepreneurs perceive towards their employees, suppliers, clients, and other stakeholders (Lechat & Torrès, 2016). Based on past research and SET, perceived economic risk (risk of bankruptcy) during the COVID-19 pandemic can be a source of stress for entrepreneurs, which can lead to burnout. Thus, we propose the following hypothesis:

- H4: The perception of economic risks is positively connected to the perception of burnout in entrepreneurs.

### Controls

We use a series of twelve control variables, eight of which pertain to the entrepreneur (age; gender; level of education; entrepreneurial experience; weekly workload; ownership stake; feelings of loneliness; pre-COVID-19 life satisfaction) and four that pertain to the business (size in terms of employees; growth of turnover; being in business because of opportunity and/or necessity). Below, we list some literature about their expected links with burnout. We address precise measurement issues in Section 3.3.

Ben Tahar (2014) pointed to various studies reporting a negative link between *age* and the development of burnout symptoms, such as Cordes and Dougherty (1993) and Maslach et al. (1996).

Several studies have shown higher levels of burnout for *women* than for men (Ben Tahar, 2014; Ben Tahar & Torrès, 2013; Lechat & Torrès, 2016). Recent meta-analyses have confirmed these gender differences, with women showing higher levels concerning the emotional exhaustion dimension of burnout (Canazei et al., 2018; Purvanova & Muros, 2010). The literature reports various reasons why women may reveal a higher level of burnout; it may have to do/go together with higher levels of “emotional exposure” (Coutrot, 2016: p. 15), worse sleep quality (Canazei et al., 2018), higher levels of insomnia (Hohagen et al., 1993), and higher levels of daytime sleepiness (Guiliani, 2016: p. 282).

Some studies have shown that *level of education* has a negative effect on burnout, probably due to easier access to medical assistance or higher levels of anticipatory behavior (Bährer-Kohler, 2013: p. 2; Ben Tahar & Torrès, 2013).

Studies by Fernet et al. (2016), Ben Tahar (2014), and Ben Tahar and Torrès (2013) have shown a positive link between *entrepreneurial experience* and burnout. This link is the combination of the effects of an increase in lassitude, a decrease in enthusiasm, and an escalation of commitment. In our study, experience is measured in years and not by some form of intensity, which makes a case for the dominant influence of lassitude and enthusiasm and a lower level of influence of escalation of commitment. This would also explain the positive link reported in the above studies.

Concerning the link with *workload* (work hours per week), there is a positive link with the risk of burnout observed both for workers in general (Maslach & Leiter, 1997) and entrepreneurs (Voltmer et al., 2011; Wei et al., 2015).

Regarding the *ownership stake*, we could not find studies dealing with its link with burnout except Lechat and Torrès (2016), who argued that an increase in financial involvement generates additional stressors,^6^ and Soenen et al. (2019), who found a link between entrepreneurial burnout and firm performance.

Several studies have pointed to the importance of *loneliness* for entrepreneurs (Akande, 1994; Gumpert & Boyd, 1984), while others have linked loneliness with health (House et al., 1988) and burnout (George-Levi et al., 2020) for the general population. Specifically, Fernet et al. (2016) showed the negative link between loneliness in the workplace and the risks of burnout for entrepreneurs.

For the general population, a positive link exists between *life satisfaction* and perceptions of good health and longevity (Diener, 1984; Diener et al., 2005; Diener & Chan, 2011), which would imply a negative connection with burnout risk. The investigation of the link between burnout and life satisfaction is also not unknown in the literature (see Hombrados-Mendieta and Cosano-Rivas (2013) for findings regarding social workers and Wang et al. (2019) for findings regarding Chinese undergraduate medical students). However, we did not find any studies showing the link between life satisfaction and the burnout of entrepreneurs.

We also consider the *size* of the business (in terms of employees) and the *growth* of turnover, and their possible relationship with burnout (Hatak et al., 2015; Soenen et al., 2019). The seminal work of Mintzberg (1979) made a connection between business size and stressors. Entrepreneurs with employees experience higher levels of distress (Godin et al., 2017; Hessels et al., 2017) than those without distress; however, Stephan (2018) reported mixed size effects.

Finally, the entrepreneurship literature routinely distinguishes between *opportunity-driven* entrepreneurs who are pulled into entrepreneurship out of choice and *necessity-driven* entrepreneurs who are pushed into starting a business for whatever reason (Reynolds et al., 2002; Van der Zwan et al., 2016). This distinction originates from early research by the Global Entrepreneurship Monitor (Bosma & Harding, 2007). The motives why entrepreneurs start their business may also impact their entrepreneurial process, as well as their health. Binder and Coad (2013) reported that there is a positive link between health and opportunity entrepreneurship but a negative link between health and necessity entrepreneurship. In our surveys, the opportunity/necessity nexus pertains to the current motives of entrepreneurs rather than to their motives when setting up their business. We suggest that the motive for being in a business in the first place will affect how entrepreneurs cope with a situation of high uncertainty and isolation, such as the COVID-19 pandemic lockdown (Durodié, 2020), which will, in turn, impact their risk of burnout (Yildirim & Solmaz, 2020).

## Data and variables

### Data: averages over time

The *Observatoire Amarok* has a long tradition of conducting surveys on entrepreneurs’ mental and physical health. 25 surveys were conducted between 2011 and 2020. Our analyses use a total of seven surveys that were conducted by *Observatoire Amarok* in the period between 2018 and 2020. These surveys are documented in Table 1. Five surveys were collected in 2018 and 2019 before the COVID-19 pandemic; these surveys offer a total of more than 1700 observations. There is a minor overlap between these five surveys that is impossible to correct due to privacy (no labels).^7^ Two surveys were collected in April and May 2020, which offer a total of nearly 3300 observations. Cronbach’s alpha value of the crucial variable BMS-10 is consistently at approximately 0.90.

**Table 1:** Seven surveys on French entrepreneurs’ burnout using BMS-10 in the 2018–2020 period

Sample number	Identifier	Coverage	Period	Respondents	Businesses of respondents	Number of respondents	BMS-10 total burnout, 10 items	Percentage BMS score ≥ 4	Percentage BMS score ≥ 5.5
	Client/name			% women, average age in years, average experience in years	% self-employed, average number of employees	Population; respondents	Average score (SD), Cronbach’s alpha		
1	Harmonie Mutuelle/VYV/C PME	France, all sectors without agriculture	2018 26 February–13 May	35.7; 50.72; 14.56	24.7; 13.11	145 K; 493	3.05 (1.03); 0.93	26.6	5.3
2	Harmonie Mutuelle/VYV/C PME	France, all sectors without agriculture	2018 7 June–20 August	33.8; 50.87; 14.38	24.7; 15.76	145 K; 296	3.05 (1.32); 0.93	28.4	4.7
3	Harmonie Mutuelle/VYV/C PME	France, all sectors without agriculture	2018 15 October–27 December	37.0; 51.34; 14.5	24.3; 13.87	145 K; 341	2.91 (1.26); 0.92	21.7	3.2
4	Harmonie Mutuelle/VYV/C PME	France, all sectors without agriculture	2019 27 March–7 June	34.64; 49.06; 11.90	26.78; 15.88	145 K; 280	2.87 (1.05); 0.90	18.2	1.4
5	Harmonie Mutuelle/VYV/C PME	France, all sectors without agriculture	2019 17 October–29 November	39.9; 50.03; 13.07	28.3; 12.16	145 K; 321	3.12 (1.62); 0.90	22.1	3.7
6	Enquête Nationale COVID-19 of 2020	France, All sectors without agriculture	2020 15 April–21 April	38.56; 49.32; 12.36	39.39; 5.55	46 K; 2899;	3.38 (1.42); 0.91	34.4	9.2
7	Enquete Covid Amarokien	France, All sectors	2020 28 April–11 May	41.16; 59.42; 16.72	28.13; 12.88	1707; 396	3.23 (1.28); 0.90	30.1	4.5

Note: BMS-10 is the Burnout Measure Short version; *sd*, standard error; France is meant to exclude Corsica and overseas territories (DOM TOM); all sectors excluding agriculture

### Data: individual data during the 2020 pandemic

For our analysis of the perception of individual burnout levels, we used two data sets of French entrepreneurs that were collected in the April–May period of 2020. The general lockdown in France was active from 17 March through 11 May 2020. Hence, both surveys were conducted during the lockdown.

The first data set is a comprehensive sample of French entrepreneurs. This sample is based on the so-called Enquête Nationale COVID-19 of 2020. The data come from a proprietary online survey and were collected from 15 April to 21 April 2020, by the research institute *Observatoire Amarok* which is connected to the University of Montpellier and Montpellier Business School, France. The survey was conducted in French and uses measures that were translated into French and validated in prior research (Lourel et al., 2007). The survey was initially sent to 46,220 entrepreneurs via e-mail. These 46,220 businesses were drawn by the Chambers of Commerce and Industry and the Chambers of Handicraft from a population of all French businesses (approximately 3.2 million). 2899 entrepreneurs participated in the survey, yielding a response rate of 6.3%. After removing the incomplete responses, this number was reduced to 2303 responses, which represents the final sample used in our main analysis.^8^ Data on the representativeness in various dimensions, such as the percentage of women, age, regions, and sectors, are available from the authors.

A smaller sample was taken during the same initial weeks of the pandemic, which we use to internally replicate our results. This sample is based on the Enquête Nationale COVID-19 of 2020 *Amarokiens*. The data come from a proprietary online survey and were collected from April 28 to May 11 in the same fashion as the Enquête Nationale COVID-19 of 2020. The survey was sent to the 1707 panel members of *Observatoire Amarok*; a total of 396 responded, yielding a response rate of 23%. After removing the incomplete responses, this number was reduced to 372 responses, which represents the final sample used in our main analysis. Representativeness has not been explicitly secured for this wave; however, as seen from Table 1, the differences between the Enquête Nationale COVID-19 of 2020 and the *Enquête COVID-19* (*2020*) *Amarokiens* in terms of percentage women, age, and experience are minimal.

### Variables

In this section, we provide a concise description of burnout, factors connected to burnout, and controls. Table 2 provides a more precise overview of the variables and definitions of the Enquête Nationale COVID-19 of 2020.

**Table 2:** Variables and definitions of the Enquête Nationale COVID-19 of 2020

Variable	Definition	Item	Scale
Dependent variable			
Burnout	Respondent’s emotional exhaustion (Burnout measure short version, BMS-10), adapted from Malach-Pines (2005)	“When you think about your work overall, how often do you feel the following?” (1) “Tired,” (2) “Disappointed with people,” (3) “Hopeless,” (4) “Trapped,” (5) “Helpless,” (6) “Depressed,” (7) “Physically weak/lickly,” (8) “Worthless/like a failure,” (9) “Difficulties sleeping,” (10) “I’ve had it”	1 = never to 7 = always
Independent variables			
Risk of COVID-19 infection	Respondent’s risk of contracting COVID-19, adapted from Jeleva (2005).	“In your opinion, how likely could you be infected with this coronavirus in the next three months?” and “In your opinion, how likely could you become seriously ill if you become infected with this coronavirus in the next three months?”	From 0 to 100%
Effect of lockdown change in life satisfaction	Difference between the respondent’s life satisfaction before and after COVID-19		From −10 to +10
Risk of bankruptcy	Bankruptcy risk of the respondent’s business, adapted from Bah and Gaillon (2016)	“When you are thinking about your company right now, what is your probability of filing for bankruptcy at the end of this crisis?”	From 0 to 100%
Controls: individual characteristics			
Age	Categorical variable that captures the respondent’s age	“What is your age?”	1 = 30 or less, 2 = 30 to 39, 3 = 40 to 49, 4 = 50 to 59, 5 = 60 or more
Female	Dummy variable that captures the respondent’s gender	“Are you female or male?”	1 = female, 0 = male
Education	Categorical variable that captures the respondent’s highest level of education	“What is your highest level of education?”	1 = none/self-taught, 2 = professional studies certificate, 3 = baccalaureate, 4 = undergraduate degrees, 5 = postgraduate degree or higher
Entrepreneurship experience	Categorical variable that captures the entrepreneurship experience via the years of business ownership	“How long have you been an entrepreneur and/or business owner?”	1 = less than 3 years, 2 = 3 to 5 years, 3 = 5 to 10 years, 4 = 10 years to 20 years, 5 = more than 20 years
Workload	Categorical variable that captures the respondent workload (hours per week)	“How many hours did you work in the previous week?”	1 = less than 40 h, 2 = 40 to 50 h, 3 = 50 to 60 h, 4 = 60 to 70 h, 5 = more than 70 h
Ownership stake	Captures the respondent’s ownership stake in their current business	“What percentage of the company’s capital do you own?	From 0 to 100%
Loneliness	Categorical variable that captures the respondent’s feeling of loneliness	“In the past month, in your job position as a business owner, did you feel …?”	1 = very surrounded, 2 = somewhat surrounded, 3 = neither lonely nor surrounded, 4 = somewhat lonely, 5 = very lonely
Life satisfaction (pre-COVID-19 and during COVID-19)	Categorical variable that captures the respondent life satisfaction prior to the COVID-19-induced confinement	“Overall, were you satisfied with the life you led before confinement?” “Overall, are you satisfied with the life you lead during confinement?”	1 = totally dissatisfied to 10 = totally satisfied
Controls: business characteristics			
Employees	Categorical variable that captures the number of employees in the company	“What is your company’s workforce, including yourself?”	1 = 0 employees, 2 = 1 to 4 employees, 3 = 5 to 9 employees, 4 = 10 to 20 employees, 5 = 20 to 49 employees, 6 = more than 60 employees
Growth of turnover (pre-COVID-19)	Categorical variable that captures the growth turnover of the respondent’s business before the COVID-19 pandemic	“Until February 2020, your growth turnover was …?”	1 = declining heavily (more than - 25%), 2 = declining (−5 to −25%), 3 = stable (± 5%), 4 = growing (from 5 to 25%), 5 = growing heavily (more than 25%)
Opportunity	Dummy variable that captures the respondent is an opportunity entrepreneur.	“Did you found your business because you wanted to seize an opportunity or because you had no other choice”?	1 = seize an opportunity, 0 = otherwise
Necessity	Dummy variable that captures the respondent is a necessity entrepreneur.	“Did you found your business because you wanted to seize an opportunity or because you had no other choice”?	1 = no choice without "..." 0 = otherwise

Note: The questionnaire was developed and conducted in French. The French version of the questionnaire is available from the authors upon request. Data was collected from April 15 to April 21, 2020, via an online survey

#### Dependent variable: burnout

We captured entrepreneurs’ perceived levels of burnout with the widely used Burnout Measure Short version (BMS-10) proposed by Malach-Pines (2005). Specifically, we used the validated translation provided by Lourel et al. (2007). This unidimensional construct represents the individual’s level of emotional exhaustion and is composed of 10 items. Cronbach’s α = 0.91 and 0.90 for the two samples used in the analyses of individual differences. An example of a question is “How often do you feel tired?” All the items are measured on a seven-point Likert scale ranging from 1 = “never” to 7 = “always.” In line with prior research, we used the mean value in our analyses (Malach-Pines, 2005).

There are various burnout measurement scales, such as the Burnout Measure (Pines & Aronson, 1988); its simplified version, the BMS-10 (Malach-Pines, 2005); the Copenhagen Burnout Inventory (Kristensen et al., 2005); the Shirom Melamed Burnout Measure (Shirom & Melamed, 2006); and the Oldenburg Burnout Inventory (Demerouti et al., 2001). We chose the BMS-10 because it is relatively short, unidimensional, and widely used. Additionally, in terms of self-observation and prevention, the BMS-10 shows good results (Carod-Artal & Vázquez-Cabrera, 2013). Using a 7-point Likert scale for the 10 items, the BMS-10 discriminates between three stages: an average below 4 indicates no burnout, an average between 4 and 5.5 indicates a mild form of burnout, and an average of 5.5 or higher signals the need for therapy.

#### Independent variables and their proxies

We measured the entrepreneurs’ health risk via the subjective likelihood of contracting COVID-19. We derived this measure from the established life expectancy construct introduced by Jeleva (2005). Our construct comprised two items: “In your opinion, how likely could you be infected with this coronavirus in the next three months?” and “In your opinion, how likely could you become seriously ill if you become infected with this coronavirus in the next three months?” The correlations are 0.55 and 0.62 for the two samples used in the analyses of individual differences. The responses range from 0 to 100%. We used the mean value of both items in our analyses.

We used the difference between life satisfaction during the COVID-19 crisis and the individual’s impression of how it was before the crisis as a measure of the lockdown risk. The questions were framed in such a way that there was a specific connection between perception of life satisfaction and the occurrence of the pandemic, thereby making this difference a perfect proxy for the lockdown effect. In this study, we were inspired by the work of Bourdeau-Lepage (2020) which focused on the entire population. This approach also allowed us to measure positive (as well as a negative) lockdown effect for entrepreneurs. For our two samples, the percentages of entrepreneurs who responded that they are experiencing a higher level of life satisfaction before than during the advent of the pandemic are 85.4 and 53.2. The responses range from −10 to 10.

We used the entrepreneur’s perception of the likelihood of bankruptcy as a measure of financial risk, which we established via the item “When you are thinking about your company right now, what is your probability of filing for bankruptcy at the end of this crisis?” The responses ranged from 0 to 100%.

#### Control variables

We included twelve control variables that referred to the respondents’ individual characteristics and the business characteristics of their venture.^9^ We did so to limit the omitted variable bias and to check the credibility of our results.

##### Individual characteristics

We included the respondent’s age to control for age-related differences between respondents. This variable is categorical and scaled from [1] “less than 30 years old” to [5] “60 years old and more.” To control for gender-related differences, we included a dummy variable that takes a value of [1] for female respondents and [0] for male respondents. We captured the respondent’s educational level via a categorical variable that scaled from [1] autodidact (i.e., no education) to [5] university-level education (equivalent to 4 years or more of education after high school). The workload (weekly number working hours) of the entrepreneur was measured using a scale ranging from [1] “less than 40 hours” to [5] “more than 70 hours.” Next, we captured the respondent’s entrepreneurship experience via a categorical variable that recorded the number of years that the respondent has owned a business. The variable ranged from [1] “less than 3 years” to [5] “more than 20 years.” Relatedly, we accounted for the respondents’ financial involvement in their business by measuring their ownership stake on a scale ranging from 0 to 100%. Furthermore, we controlled for two psychological variables that could impact the perceived level of burnout. First, we captured the respondent’s feeling of loneliness on a five-point scale that ranged from [1] “very surrounded” to [5] “very lonely.” Second, we captured the respondent’s life satisfaction before the COVID-19 pandemic on a 10-point scale ([1] “totally dissatisfied” to [10] “totally satisfied”) via the question “Overall, were you satisfied with the life you led before confinement?”

##### Business characteristics

To account for a potential relationship between business characteristics and the respondent’s perceived level of burnout, we controlled for the size of the respondent’s business via the number of employees. The categorical variable was scaled from [1] “0 employees” (i.e., solo-entrepreneur) to [6] “50 employees and more.” Next, we captured the venture’s business development via the growth or decline in turnover shortly before the COVID-19 pandemic (“Until February 2020, your growth turnover was…?”). The responses ranged from [1] “declining heavily (more than −25%)” to [5] “increasing significantly (more than 25%).” Finally, we captured whether the respondent is an opportunity entrepreneur or a necessity entrepreneur. The questions were adapted from the Global Entrepreneurship Monitor (Reynolds et al., 2002). We constructed two dummy variables that measured whether the entrepreneur feels that she is in the business because she wants to/because of an opportunity ([1] opportunity, [0] otherwise) or whether the entrepreneur feels that there is no satisfactory choice ([1] necessity, [0] otherwise).

All variables and their definitions are summarized in Table 2 for our main dataset, which is the Enquête Nationale COVID-19 of 2020.

## Results and analysis

First, we will address the average perception of burnout over time using seven data sets and then with individual burnout levels during the pandemic using two data sets.

### Average burnout over time

Table 1 tells a clear story irrespective of the statistic chosen. The average burnout score is higher in surveys 6 (Enquête Nationale COVID-19 of 2020) and 7 (*Enquête COVID-19* (*2020*) *Amarokiens*) than in pre-COVID-19 surveys 1 to 5 (*Harmonie Mutuelle*). The same can be concluded for the percentage BMS score ≥ 4 (indicating a mild form of burnout) and for the BMS score ≥ 5.5 (indicating the need for therapy). These increases are significant considering the number of observations. These findings support H1.

### Individual burnout during the crisis

#### Descriptive results of the Enquête Nationale COVID-19 of 2020

##### Dependent variable

The average level of burnout among our 2336 respondents is 3.39.

##### Independent variables

On average, the respondents assumed that their risk of contracting COVID-19 in the following 3 months after the survey (i.e., May, June, and July of 2020) was 35%, while the average risk of going bankrupt due to the COVID-19 pandemic was estimated to be 30%. The effects of the lockdown (measured in terms of the degradation of life satisfaction) are also perceived to be positive. The correlations between these three factors are relatively small, while the between infection and lockdown is practically zero.

##### Control variables: individual characteristics

The largest age group among our respondents was between 50 and 59 years old (38.7%), followed by the group of respondents between 40 and 49 years old (29.9%) and the group of respondents 60 years old or older (14.1%). The majority (63.1%) of our respondents were male, had a university-level education (53.3%), and had owned a business for more than 10 years (54.6%). In addition, 62.3% of our respondents had an ownership stake of 100% in their venture, suggesting high financial involvement. In contrast, 6.7% indicated that their ownership stake is below 25%. The average feeling of loneliness reported by our respondents was slightly above the average, with a value of 3.32 (5-point scale). The average life satisfaction before the COVID-19 pandemic was assessed positively, with an average value of 7.63 (10-point scale).

##### Control variables: business characteristics

The majority of our respondents were the owners of small firms: 67.2% of them indicated that their ventures have one to five employees, while only 1.7% have more than 50 employees. Concerning the growth of turnover before the COVID-19 pandemic (i.e., in February 2020), most respondents indicated that their businesses had been stable (46.0%) or had experienced positive growth rates of 5% or more (43.9%). Finally, 77.1% of the respondents indicated that they started their business to pursue an opportunity, while 33.0% indicated that they started their business out of necessity. Note that the respondents could select both options simultaneously.

Descriptive statistics, correlations, and variance inflation factors (VIFs) are displayed in Table 3. The low VIF values indicate that multicollinearity does not severely bias our results.

**Table 3:** Descriptive statistics, correlations, and variance inflation factors (VIFs) of the Enquête Nationale COVID-19 of 2020

Variable	Mean	SD	Min	Max	1	2	3	4	5	6	7	8	9	10	11	12	13	14	15	VIF
Dependent variable																				
1. Burnout	3.39	1.42	1	7																1.63
Independent variables																				
2. Risk of COVID-19 infection	35.33	22.40	0	100	0.23*															1.09
3. Effect of lockdown (change in life satisfaction)	3.67	2.78	−7	10	0.20*	0.03														1.59
4. Risk of bankruptcy	29.85	28.53	0	100	0.47*	0.19*	0.18*													1.51
Controls: individual characteristics																				
5. Age	3.47	0.99	1	5	−0.10*	−0.01	0.03	−0.01												1.30
6. Female	0.37	0.48	0	1	0.09*	0.00	−0.02	0.04	−0.11*											1.11
7. Education	3.46	1.18	1	5	−0.07*	−0.07*	−0.14*	−0.05	−0.04	0.07*										1.09
8. Entrepreneurship experience	3.38	1.34	1	5	−0.02	−0.03	−0.04	−0.06*	0.44*	−0.15*	−0.08*									1.33
9. Workload	1.56	1.00	1	5	0.00	0.02	−0.13*	−0.12*	0.04	−0.16*	0.12*	0.10*								1.16
10. Ownership stake	4.14	1.27	1	5	0.01	0.05	0.06*	−0.01	−0.01	−0.04	−0.05	−0.04	−0.01							1.06
11. Loneliness	3.32	1.16	1	5	0.35*	0.09*	0.13*	0.33*	−0.01	−0.05	−0.01	−0.06*	−0.04	0.01						1.23
12. Life satisfaction	7.62	1.67	0	10	−0.28*	−0.06*	0.44*	−0.27*	0.00	−0.05	−0.01	−0.01	−0.04	0.07*	−0.16*					1.66
Controls: business characteristics																				
13. Employees	2.49	0.93	1	6	−0.05*	−0.09*	−0.04	−0.20*	0.07*	−0.18*	0.16*	0.22*	0.31*	−0.19*	−0.11*	0.04				1.30
14. Growth of turnover	3.39	0.80	1	5	−0.09*	−0.05	0.08*	−0.13*	−0.12*	−0.05	0.07*	−0.09*	0.02	−0.02	−0.03	0.18*	0.08*			1.08
15. Opportunity	0.77	0.42	0	1	0.00	−0.03	0.00	−0.03	−0.15*	0.00	0.03	−0.08*	0.00	0.01	−0.04	0.06*	0.03	0.03		1.06
16. Necessity	0.33	0.47	0	0	0.14*	0.08*	−0.03	0.11*	0.07*	0.03	−0.04	0.02	0.00	0.01	0.11*	−0.16*	−0.05	−0.07*	−0.18*	1.08

Note: * *p* < 0.01. VIFs are estimated based on model 4

#### Regression analysis

To explain burnout levels during the COVID-19 crisis and to test our hypotheses, we performed a multivariate OLS regression analysis that used the respondent’s level of perceived burnout as the dependent variable. The results are displayed in Table 4. Since we were particularly interested in discriminating between health risks (the threat of becoming ill, H2), the effects connected to the lockdown (the degradation of life satisfaction, H3), and the risks connected to finance (the threat of having to file for bankruptcy, H4), we added our dependent variables in a stepwise manner. While model (1) only included the control variables, the independent variables were entered stepwise in models (2), (3), and (4). Our following explanations and interpretations refer to model (5), in which all three risk variables were entered simultaneously.

**Table 4:** Main analysis: OLS regression analysis with “burnout” as the dependent variable of the Enquête Nationale COVID-19 of 2020

Model	(1)	(2)	(3)	(4)	(5)
Variables	Coeff. (SE)	Coeff. (SE)	Coeff. (SE)	Coeff. (SE)	Coeff. (SE)
Controls: individual characteristics					
Age	−0.173 (0.030)***	−0.172 (0.029)***	−0.197 (0.028)***	−0.176 (0.028)***	−0.192 (0.027)***
Female	0.288 (0.057)***	0.289 (0.056)***	0.283 (0.054)***	0.290 (0.053)***	0.286 (0.050)***
Education	−0.089 (0.023)***	−0.076 (0.023)***	−0.036 (0.022)	−0.081 (0.021)***	−0.034 (0.021)*
Entrepreneurship experience	0.050 (0.023)**	0.054 (0.022)**	0.068 (0.021)***	0.058 (0.021)***	0.072 (0.020)***
Workload	0.034 (0.028)	0.020 (0.028)	0.082 (0.027)***	0.075 (0.026)***	0.090 (0.025)***
Ownership stake	0.023 (0.021)	0.017 (0.021)	0.018 (0.020)	0.036 (0.020)*	0.024 (0.019)
Loneliness	0.396 (0.023)***	0.381 (0.023)***	0.312 (0.023)***	0.273 (0.023)***	0.231 (0.022)***
Life satisfaction (pre-COVID-19)	−0.179 (0.017)***	−0.174 (0.016)***	−0.316 (0.017)***	−0.116 (0.016)***	−0.229 (0.017)***
Controls: business characteristics					
Employees	0.033 (0.032)	0.054 (0.031)*	0.021 (0.030)	0.105 (0.030)***	0.092 (0.028)***
Growth of turnover (pre-COVID-19)	−0.062 (0.034)*	−0.053 (0.033)	−0.071 (0.032)**	−0.011 (0.032)	−0.024 (0.030)
Opportunity	0.098 (0.064)	0.104 (0.063)	0.105 (0.061)*	0.096 (0.060)	0.105 (0.057)*
Necessity	0.239 (0.058)***	0.204 (0.057)***	0.228 (0.055)***	0.195 (0.054)***	0.172 (0.051)***
Independent variables					
Risk of COVID-19 infection		0.012 (0.001)***			0.009 (0.001)***
Effect of lockdown (change in life satisfaction)			0.179 (0.010)***		0.132 (0.010)***
Risk of bankruptcy				0.019 (0.001)***	0.014 (0.001)***
Observations	2.297	2.297	2.297	2.297	2.297
R^2^	0.209	0.247	0.303	0.324	0.383
R^2^ (adj.)	0.213	0.243	0.299	0.321	0.387

Note: * *p* < 0.10, ** *p* < 0.05, *** *p* < 0.01

With regard to H2, the results of our regression analysis show that higher levels of burnout correspond with a higher risk of infection and illness (*p* < 0.01). In line with H2, higher levels of burnout are also associated with higher perceived effects of lockdown (*p* < 0.01). Finally, higher levels of burnout are associated with a higher risk of bankruptcy (*p* < 0.01), which is in line with H3. In summary, all three effects are highly significant and lend support to our hypotheses.

In addition, several control variables affect the respondent’s level of burnout in a statistically significant way. Among the individual characteristics, a positive relationship exists between higher levels of burnout and being female (*p* < 0.01), higher levels of entrepreneurship experience (*p* < 0.01), higher ownership stakes (*p* < 0.10), and higher levels of loneliness (*p* < 0.01). In contrast, a negative relationship exists between higher levels of burnout and age (*p* < 0.01), education (*p* < 0.01), and respondents’ life satisfaction before the COVID-19 pandemic. Concerning business characteristics, a positive relationship exists between the level of burnout and the number of employees in the respondent’s business (*p* < 0.01). Additionally, those who responded that they were entrepreneurs because of opportunities (*p* < 0.10) or out of necessity (*p* < 0.01) experience higher levels of perceived burnout than the respondents who did not report these motives. By and large, the signs of the coefficients of the controls are in line with the literature and our predictions, as dealt with in Section 2. This outcome provides credibility in our outcomes.

Comparing models (2) through (5), we conclude that the influence of the three factors is largely independent of each other (comparing coefficients across the models) and that the risk of bankruptcy has the highest effect on burnout, while the risk of infection the lowest effect on burnout (by comparing differences in R^2^ across the models).

To assess the robustness of our main findings, we perform a replication using an additional dataset of 372 individuals collected during the same period. The results are reported in Table 5. Despite the smaller sample size, this assessment confirms the results of our independent variables with regard to significance and magnitude. The risk of COVID-19 infection and illness (*p* < 0.01), the effects of the lockdown (*p* < 0.01), and the risk of bankruptcy (*p* < 0.01) affect the respondents’ levels of burnout positively and in a statistically significant way.

**Table 5:** Replication: OLS regression analysis with “burnout” as the dependent variable using the *Enquête COVID-19 (2020) and the Enquête COVID-19 (2020) Amarokiens*

Model	(1)	(2)
Description	Main analysis	Replication
	*Enquête Nationale COVID-19 of 2020*	*Enquete Covid Amarokiens*
Variables	Coeff. (SE)	Coeff. (SE)
Controls: individual characteristics		
Age	−0.192 (0.027)***	−0.185 (0.071)***
Female	0.286 (0.050)***	0.318 (0.114)***
Education	−0.034 (0.021)*	0.002 (0.055)
Entrepreneurship experience	0.072 (0.020)***	0.154 (0.058)***
Workload	0.090 (0.025)***	0.117 (0.049)**
Ownership stake	0.024 (0.019)	−0.065 (0.047)
Loneliness	0.231 (0.022)***	0.241 (0.049)***
Life satisfaction (pre-COVID-19)	−0.229 (0.017)***	−0.293 (0.036)***
Controls: business characteristics		
Employees	0.092 (0.028)***	−0.014 (0.050)
Growth of turnover (pre-COVID-19)	−0.024 (0.030)	−0.096 (0.071)
Opportunity	0.105 (0.057)*	0.120 (0.125)
Necessity	0.172 (0.051)***	0.086 (0.123)
Independent variables		
Risk of COVID-19 infection	0.009 (0.001)***	0.012 (0.003)***
Effect of lockdown (change in life satisfaction)	0.132 (0.010)***	0.212 (0.028)***
Risk of bankruptcy	0.014 (0.001)***	0.004 (0.003)*
Observations	0.383	0.371
R^2^	2.297	372
R^2^ (adj.)	0.387	0.396

Note: * *p* < 0.10, ** *p* < 0.05, *** *p* < 0.01

Finally, we performed three more robustness tests. *First*, we cannot simply assume that all small businesses are led by a single person. The fact that a firm may be led by more than one person blurs the link between burnout and outside threats. We tried to correct for the person-business discrepancy by using the ownership stake as one of the controls. However, this is a crude correction. A better analysis is running a regression for individuals who own more than 50% of the capital and comparing the results to those of a regression involving all individuals. The results of these two regression exercises are almost identical. Additionally, approximately 90% of the individuals in our sample reported owning more than 50% of their business. *Second*, both the change in life satisfaction and its level are used in the regressions. The correlation between the two is not high by definition; nevertheless, in our case, it is 0.44 (Table 3). However, there is no real danger of distortion by multicollinearity. For instance, the coefficient of change of life satisfaction (the proxy for lockdown effects) in Table 5 model 1 stays significant even when excluding life satisfaction (control), and the value is 0.06 (0.009)***. Third, in addition to our present OLS analysis, we also performed a SEM analysis. Given the linear structure of our analysis, with no assumptions about mediating effects, no drastic differences were expected. Indeed, all the coefficients are similar between the OLS and SEM approaches for both samples, except for those of workload.

## Discussion and conclusion

Economic crises negatively impact workers’ mental health (Mucci et al., 2016). The economic damage of the COVID-19 pandemic is predicted to be severe, ubiquitous, and long-lasting. Other than those who were directly affected by the virus due to their own illness as well as the illness of their relatives, friends and colleagues, there is a host of economic actors who feels threatened,  due to both health and economic reasons.

When writing the present paper (winter 2020/21), the contours of public health and the economic effects of COVID-19 were slowly but inevitably becoming clear.^10^ Economic adversity can have many facets, out of which (the threat of) unemployment is probably the worst. There is bound to be a serious effect on mental health caused by the traumatic experiences of the pandemic. COVID-19-related stress and other impairments may be the result. (See Hossain et al. (2020) and Odriozola-González et al. (2020) for some very early opinions and results.)

Recently, a study investigated the impacts of the COVID-19 pandemic on stress and burnout in the general population (Yildirim & Solmaz, 2020). To our knowledge, no empirical studies have investigated these links in entrepreneurs.^11^ When entrepreneurs experience an increased level of stress, their level of burnout is impacted (Fernet et al., 2016; Wei et al., 2015). Moreover, work stress has been identified as the principal cause of entrepreneurial burnout (Omrane et al., 2018). Taken together, COVID-19-related stress may impact the burnout of entrepreneurs because this new source of stress may be an additional factor in their pre-existing daily stress (Lechat & Torrès, 2016; Wach et al., 2020).

The present paper investigates if the levels of burnout among French entrepreneurs indeed increased during the first weeks of the COVID-19 pandemic (from what it was before) and whether the variation in these burnout levels is linked to their perception of the threat of becoming ill due to the pandemic, the effects of having to stay at home due to the lockdown and/or the threat of having to file for bankruptcy due to the economic downturn.

We focus on entrepreneurs because, on the one hand, they have been severely hit by the COVID-19 crisis (Bartik et al., 2020; Beland et al., 2020; Block et al., 2021; Fairlie, 2020; Meahjohn & Persad, 2020; Thorgren & Williams, 2020), while, on the other hand, the surviving incumbent ones (as well as the new ones) are expected to play a crucial role in the recovery of economic disasters (Eggers, 2020; Morrish & Jones, 2020; Zvikaramba et al., 2020). By recovery, we do not simply mean the reconstruction of the pre-COVID-19 economy but rather an economy with a higher emphasis on existing long-term goals (such as lower carbon dependence (Markard & Rosenbloom, 2020)) and on new COVID-19-related wisdom (such as investment in health care and medical technologies and services, investment in local initiatives instead of international supply chain components, investment in new business models in the hotel and catering industry, and investment in gadgets making distance communication easier).

Research indicates that there are certain job demands specific for entrepreneurs based on their job characteristics (as compared to other salaried workers): time demands, uncertainty, risk, responsibility (Dijkhuizen et al., 2014; Gorgievski & Laguna, 2008), for which entrepreneurs are likely to experience a higher level of stress than their salaried counterparts. With the advent of the pandemic, the demands on entrepreneurs have been accentuated (as we note in Section 2.1). Thus, we can argue that the stress level of entrepreneurs has increased in comparison to, for example, salaried workers (many of whom still draw a paycheck at the end of the month).

This argument is congruent with the theoretical framework of the Stress Events Theory (Lerman et al., 2020), the Conservation of Resource Theory (Hobfoll, 1989), and the Job-Demand-Control Model (Demerouti et al., 2001) as delineated in the hypotheses development section of the current paper. For entrepreneurs (who shoulder the primary responsibility of their business), the uncertainty and risk ushered by the pandemic are much more consequential. Thus, we believe that our empirical study on entrepreneurs conducted during the COVID-19 pandemic indeed allows for a unique contribution to the literature on entrepreneurs. We used two samples that were collected at the advent of the pandemic to provide some replication power to our outcomes, and we compared their outcomes with those of several samples that were collected before the advent of COVID-19.

There are two main conclusions drawn from our study. Our *first* conclusion is that the average burnout levels using the BMS-10 (Malach-Pines, 2005) for the two samples taken during the COVID-19 pandemic are higher than those for the five earlier samples. The differences are significant considering the number of sample observations. In a follow-up study, Torrès, Benzari, et al. (2021b) go deeper into the evolution of the ten items of the BMS-10 over time. They observe that the increase of the global BMS-10 measure is mainly due to the considerable increase of the items “être coincé” (feeling trapped) and “impuissance” (helplessness).^12^

Our *second* conclusion is that perceived levels of burnout of French entrepreneurs during the COVID-19 crisis are linked to all three factors investigated in the present paper, namely, the risk of becoming ill (contracting the virus), the effects associated with having to stay at home due to the lockdown, and the risk of having to file for bankruptcy due to the economic downturn. The effects of the latter two factors are larger than those of the former, while the bankruptcy risk is the largest. The (bilateral) contributions in percentage points to the R^2^ of our OLS regression are 3.4, 9.0, and 11.1 for the risk of becoming ill, the effects associated with having to stay at home, and the risk of bankruptcy, respectively. It seems that when having to choose between their own personal health and the health of their business, entrepreneurs are focused upon the latter. Moreover, since the perceived negative effects connected to the lockdown are considerable and higher than those of the risk of becoming ill, this is a signal to the authorities to be extremely careful about applying general lockdowns.

A final point worth mentioning is the signs of the coefficients of the many controls we use for the analysis of the links between the risk of burnout and the three independent variables (illness, lockdown, and bankruptcy). These coefficient signs are mainly in line with our expectations in both data sets, which provides credibility for our findings.

Our study contributes to the research on mental health in entrepreneurs (Omrane et al., 2018; Stephan, 2018; Torrès & Thurik, 2019) and, more specifically, to the perceptions of burnout during the COVID-19 pandemic, which can be interpreted as a natural experiment on the level of variables and their links. Moreover, our work contributes to the literature on risk perception. Risk perception towards COVID-19 and its understanding are important topics (Van Bavel et al., 2020). There is a current call in the literature for understanding the “new vulnerabilities [of entrepreneurs] that diminish their psychological wellbeing” (Shepherd, 2020). In our study, by looking at two kinds of risk perception (while viewing it as the appraisal of danger or threat (Dryhurst et al., 2020)) for entrepreneurs during COVID-19, we show how the perception of both health and economic risks linked to COVID-19 endangers entrepreneurs’ mental health and leads to burnout risks. In addition to these two types of risks, we also investigate the effects of the lockdown on the risk of burnout. The discrimination between three possible causes of burnout, i.e., health risk, effects of lockdown, and financial risk, is a novel approach.

## Limitations and future research

The BMS-10 is one of the most widely used burnout measures. We have no reasons to assume that other measures, such as the full Burnout Measure of Pines and Aronson (1988), the Maslach Burnout Inventory (Maslach et al., 1996), the Copenhagen Burnout Inventory (Kristensen et al., 2005), the Shirom Melamed Burnout Measure (Shirom & Melamed, 2006), or the Oldenburg Burnout Inventory (Demerouti et al., 2001), would have led to different results, although we could not test this assumption empirically due to a lack of data. However, other self-reported indicators, such as quality of sleep, may have helped establish the mental health state of individuals; objective clinical measures, such as the number of visits to doctors, may have also helped shed additional light on this state. Last, we have no individual data on the perceived risk of bankruptcy before COVID-19. The other two risk factors (related to illness and lockdown) were not relevant before COVID-19. Thus, a comparison between the three is not feasible in a pre-COVID-19 setting. Nevertheless, a comparison between the perceived risk of bankruptcy before and during the COVID-19 pandemic could have strengthened our results.

The dominance of the financial effect over the health effect regarding the perception of the risk of burnout seems to point to the existential instinct of entrepreneurs to work, maintain, and develop their business. If they cannot pursue these drivers, they become frustrated and burnout looms. This frustration is also personified by what Sartre observed in his thesis of existentialism—humans are “comme la somme de ses actes” (Sartre, 2009). Indeed, for most people, work represents an existential quest that, if failed, leads to a high risk of burnout (Pines, 1993). In other words, when individuals choose a career path such as business ownership, success represents “a sense of existential significance”; however, in the face of losing this sense of meaning and the spirit of life, “failure is a powerful cause of burnout” (Pines, 1994: p. 383).^13^ Further research may dig deeper into this existentialist view and the propensity to become an entrepreneur.

Clearly, the French environment and mentality may have singularities (Beugelsdijk & Welzel, 2018) that need to be considered when testing our model in different cultures and for different mentalities, especially concerning burnout (Schaufeli, 2017). Moreover, risk perceptions are a phenomenon that should be also controlled for (Dryhurst et al., 2020).

Like all proxies, our proxy for the effect of the lockdown has shortcomings. We wanted this proxy to meet three criteria: there should be a *mental element* (because of the link with burnout); there should be a *temporal effect* (a change over time); there should be an *explicit link to the lockdown*. A change in life satisfaction based upon two separate questions referring to before and during the pandemic and also explicitly referring to the lockdown is then an obvious candidate. The proxy may be related to more than just the lockdown but the questions are asked expressly mentioning the lockdown. It may be linked to the other two risks. Table 3 shows that the correlation with the perceived infection risk is very low. The correlation with the perceived risk of bankruptcy is not low which could lead to multicollinearity. Leaving out the variable “change in life satisfaction” does not change the remaining coefficients. (See Table 4.) This of course is a good indicator that coefficients do not suffer from multicollinearity bias. This can also be concluded from the relatively low VIF (see Table 3). Taken together, including or not the proxy for the lockdown effect does not affect remaining effects. Nevertheless, multi-item proxies zooming in on the various effects of a lockdown may improve its measurement.

Finally, our study can be regarded as specific for entrepreneurs in an empirical fashion: the risk of bankruptcy is specific for entrepreneurs and many of the controls are irrelevant for non-entrepreneurs. We would think that for individuals in general, in particular, salaried people (who have a guaranteed paycheck at the end of the month), the effect of pandemic, while it may certainly be negative for their mental and physical health, would not be the same as for entrepreneurs (for who, as Mintzberg (1979: p. 312) had rightly noted “One heart attack can literally wipe out the organization’s prime coordinating mechanism”). However, risk of bankruptcy can be exchanged for risk of lay-off, appropriate independents and controls can be chosen and the entire setup can be supplanted to employees, and a comparison between entrepreneurs and salaried workers can be made. This would, however, lead to an entirely different research setup.

## Recommendations

Since our findings reveal that the perception of risk of burnout has grown significantly since the advent of the COVID-19 pandemic, we believe that any kind of support, including nonfinancial support, could be helpful at this time. A variety of efforts could be put in place. For example, support networks could be set up to prevent pathogenic effects (Torrès & Thurik, 2019). Past research has recommended setting up “anti-suicide funding” for this type of emergency/crisis situation (Bortolussi, 2012). Recently, the French government, together with non-governmental agencies, has started a special telephone rescue and support service for non-salaried workers (e.g., entrepreneurs). Webinars constitute another manner of communication, given the novelty of COVID-19-related pathogenic effects; non-salaried workers know less about the causes of mental illnesses than do salaried workers, which demands more awareness programs for these individuals (Torrès & Thurik, 2019). Along with fighting the pathogenic effects, promoting salutogenic effects is yet another way to provide support to entrepreneurs. Providing coaching services that encourage entrepreneurs to elevate their sense of life satisfaction could also be highly useful.

The strong link between loneliness and the risk of burnout is striking (see Table 5). It is a phenomenon that can be influenced in the short run as opposed to other controls such as age, gender, education, and experience. The same can also be true if an entrepreneur feels that there is no satisfactory alternative choice other than to continue running the business (the necessity effect). However, if this is indeed the case, this can be addressed through proper guidance, organized by public and private support groups, since we know that social support is one of the key factors for good health (Gunnarsson & Josephson, 2011).

The COVID-19-related increase in the perceived level of burnout among French entrepreneurs and the considerable influence of the perceived risk of bankruptcy points to the urgency of understanding and assisting with the phenomenon of burnout for the health and economic survival of these entrepreneurs. Their presumed role in the recovery of the entire economic system necessitates the setup of an “entrepreneurship care” structure that includes telephone support, webinars, and emergency services.

## Footnotes

1 In the present text, we the occupational definition of entrepreneurs as individuals who work for their own account and risk in the Hébert and Link (1989) sense. This definition includes self-employed and those employing others.

2 We recognize that the second effect may not be detrimental at all for certain professions that can be executed as well or even better without direct contact with suppliers, clients, etc. In fact, the pandemic may sometimes play a positive role due to a “sabbatical” effect (Palmer et al., 2020).

3 Obviously, there may be compensating effects dampening or magnifying these levels of burnout for industries more or less affected by the unprecedented economic slowdown. The agricultural sector and the online retail sector may be less affected (INSEE, 2020a, 2020b), while the hotel, catering, and leisure sector may be more affected (INSEE, 2020a, 2020b).

4 In fact, the JD-R model recognizes the role of “home demands” on exhaustion and burnout (Bakker et al., 2005)

5 Of course, quarantine and lockdown are not the same, but it is easy to imagine that a period of quarantine or even its threat may have similar negative effects as a period of lockdown.

6 In her section 2.4, Stephan (2018) deals with firm finance and entrepreneurial well-being.

7 Some overlap may occur between the five surveys prior to the advent of COVID-19. However, privacy regulations (Règlement Général de Protection des Données) prevent us from establishing exactly how big this overlap is because the surveys are anonymized. Close inspection of the variables enables an approximation to be made and shows that the overlap is very low, i.e., between 5.5 and 7.5% for consecutive surveys. The overlap among the first three surveys is 1.5%.

8 The *Enquête Nationale Covid* (*2020*) has also been used (with *n* = 1925 instead of *n* = 2303 in the present study) as part of a study of physical and mental perception before and during the COVID-19 pandemic. See Torrès, Swalhi, Mukerjee, Lasch and Thurik (2021a).

9 Most controls are also used as part of a study of physical and mental perception using data sets before and during the COVID-19 pandemic (Torrès, Swalhi, Mukerjee, Lasch and Thurik, 2021a).

10 See the OECD Tackling Coronavirus Series, oecd.org/coronavirus/en/.

11 In the present text, we the occupational definition of entrepreneurs as individuals who work for their own account and risk in the Hébert and Link (1989) sense. This definition includes self-employed and those employing others.

12 See Torrès et al. (2021b) for an analysis of the evolution of BMS-10 items in French entrepreneurs over time. They observe that during the pandemic, a new hierarchy of items appears with “feeling trapped” and “helplessness” as the two most important items and they conclude that this points at an unprecedented form of impediment exhaustion which they propose to call *impediment exhaustion syndrome*.

13 “Success gives highly motivated individuals a sense of existential significance and partially heals their childhood wounds. When they feel that they have failed (to do the work the way it “should” have been done), when the work does not give their life a sense of meaning, they burn out.” (Pines, 2000: p. 634).
